# Quality-Adjusted Life Years and Disability-Adjusted Life Years Are Better With Concurrent Chemoradiation Therapy Than Induction Chemotherapy Followed by Chemoradiation Therapy in Nasopharyngeal Carcinoma

**DOI:** 10.7759/cureus.13022

**Published:** 2021-01-30

**Authors:** Mary R Nittala, Madhava R Kanakamedala, Eswar Mundra, William C Woods, Maria L Smith, Robert D Hamilton, Gina D Jefferson, Lana Jackson, Satya Packianathan, Srinivasan Vijayakumar

**Affiliations:** 1 Radiation Oncology, University of Mississippi Medical Center, Jackson, USA; 2 Radiation Oncology, Texas Oncology, Amarillo, USA; 3 Hematology and Medical Oncology, University of Mississippi Medical Center, Jackson, USA; 4 Otolaryngology - Head and Neck Surgery, University of Mississippi Medical Center, Jackson, USA

**Keywords:** disability-adjusted life year, nasopharyngeal carcinoma, quality-adjusted life year

## Abstract

Introduction

As traditional measures such as overall survival (OS) or disease-free survival (DFS) alone do not give a holistic view of the outcomes of a treatment paradigm, we determine to add the evidence of quality-adjusted life year (QALY) and disability-adjusted life year (DALY) to the outcomes of the nasopharyngeal carcinoma patients (NCP) treated with definitive chemoradiation therapy (chemoRT) with or without induction chemotherapy (induction chemo).

Methods

This is a retrospective analysis of 85 NCPs treated at an academic state institution. The OS estimated by the Kaplan-Meier method and the multivariate Cox regression model determined the co-variables associated with the OS. The relationship between QALYs gained and DALYs saved were calculated from age of the disease onset, duration of the disease, quality of life (QoL) and disability weights.

Results

Of the 85 eligible NCPs of this cohort, the disease frequency distribution per the World Health Organization (WHO) classification was 41.2% for Type-I, 42.4% for Type-II, and 16.5% for Type-III. The median follow-up (24 months). The five-year OS of patients treated with concurrent chemoRT *vs*. induction chemo followed by concurrent chemoRT was 54.7 *vs*. 14.8% for WHO Type I, 60.1 *vs.* 58.3% for WHO Type II, and 83.3 *vs.* 50.0% for WHO Type III (p=0.029). The average DALYs saved with concurrent chemoRT were 12.2 years *vs.* 5 years for induction chemo followed by concurrent chemoRT. The average QALYs gained with concurrent chemoRT were 6.9 years *vs.* 3.1 years for induction chemo followed by concurrent chemoRT.

Conclusion

Patients treated with concurrent chemoRT had an increased QoL when compared to induction chemo followed by concurrent chemoRT. The average DALYs saved were higher in the patients treated with concurrent chemoRT than treated with induction chemo followed by concurrent chemoRT.

## Introduction

In the United States, nasopharyngeal carcinoma (NPC) is a rare neoplasm of the head and neck (HN) region and accounts for only 2% of all HN cancers; less than 1:10,000 persons are diagnosed with NPC each year and 129,000 new cases were diagnosed in 2018 [1,2]. Worldwide, however, it is the 18th and 22nd most commonly occurring cancer in men and women, respectively [3]. It is more common in Asian populations such as those originating from Southern China and Southeast Asia as well as in Eskimo and North African population, with annual incidence rates approaching 50 cases per 100,000 persons [4].

Nasopharyngeal carcinoma differs from other HN cancers in epidemiology, distant metastasis rates, and its association with the Epstein-Barr virus (EBV) [5]. The major source of morbidity and mortality in NPC patients comes from its high risk of distant metastasis. The World Health Organization (WHO) has classified NPC into three types based on histology: keratinizing squamous cell carcinoma (Type I), differentiated non-keratinizing carcinoma (Type II), and undifferentiated non-keratinizing carcinoma (Type III) [6,7]. In the US, about 25% of NPC patients have keratinizing carcinoma while 12% are differentiated non-keratinizing and 63% have the undifferentiated non-keratinizing histology [7].

Treatment for NPC depends upon the tumor stage and the patient's performance status [8]. Because of the operative morbidity associated with the anatomic location of these tumors, they are traditionally treated with radiation therapy (RT) [8,9]. Both non-keratinizing carcinomas (Type II & III) respond better to radiation compared to the keratinizing carcinoma (Type I). The five-year overall survival rates for non-keratinizing NPC is 51% *vs.* 6% for the keratinizing NPC [10]. Previous studies have suggested that OS rates of NPC patients were not significantly improved by induction chemotherapy (induction chemo) followed by concurrent chemoRT compared to concurrent chemoRT [11], and could even worsen the survival of stage II NPC patients [12].

A patient’s pre-treatment health-related quality of life (HRQoL) has been shown to be a significant independent predictor of locoregional disease control in HN cancer patients [13]. Thus, an understanding of the contribution of various treatment modalities to a patient’s QoL is very important as it because it helps to evaluate the type of treatment the HN cancer patients may need and also identify impairments in the ability to tolerate a specific treatment approach [14].

Traditionally, QoL is often evaluated using health survey questionnaires such as the Quality of Life Questionnaire C30 (QLQ-C30) or the European Organization for Research and Treatment of Cancer Quality of Life Questionnaire (EORTC QLQ) Head and Neck Cancer-Specific Module (H&N35) [15]. When questionnaires are unavailable, QoL is often estimated by alternative methods of quality-adjusted life year (QALY) and disability-adjusted life year (DALY) [16].

QALY captures both the impact of a treatment on a patient’s length of life and also its impact on their HRQoL [17]. DALY is a summary measure of public health widely used to quantify the burden of disease [18]. In this study, we used QALYs and DALYs to assess the impact of QoL on survival outcomes of NPC patients treated with concurrent chemoRT with or without induction chemotherapy.

This article was previously presented as a meeting abstract at the 2019 AACR-AHNS Head and Neck Cancer Conference at Austin, TX, USA on April 29, 2019. This article was previously posted to the Research Square preprint server on August 26, 2020.

## Materials and methods

Patients

The study subjects were 96 NPC patients diagnosed and treated between 1994 and 2018 at the University of Mississippi Medical Center (UMMC), Jackson, MS, USA. Institutional review board approval was obtained for the retrospective analysis. A browser-based database -- Research Electronic Data Capture (REDCap; Vanderbilt University, Nashville, TN, USA) -- was used to gather and store the patient information. Written consent was waived secondary to the retrospective nature of the study and patient identifiers were removed before the data were extracted for analysis. Nine patients with an unknown WHO class of tumor were excluded from the study and hence, 85 patients were included in the final analyses.

Treatment methods

Fifty-one patients (60%) received concurrent chemoRT consisting of weekly cisplatin (40 mg/m2) during radiotherapy for a maximum of seven cycles, beginning on the first day of radiotherapy. Twenty patients (23.5%) received induction chemo with docetaxel, cisplatin, and fluorouracil (TPF) followed by concurrent chemoRT as described above, while 14 patients (16.5%) received no treatment.

QoL assessments

QALY: Is a measure of the value of health outcome and combines length and QoL into a single unit. It is calculated simply by multiplying the duration of time spent in a health stage by the HRQoL weighting associated with that health state.

DALY: Is a common measurement unit for morbidity and mortality. It is composed of years lived with the disability (YLD) and years of life lost (YLL) due to premature mortality associated with that disability.

DALY = YLD + YLL

YLD = number of cases x duration till remission (or) death x disability weight (DW)

YLL = number of deaths x life expectancy (LE) at the age of death

DW is a scale from zero (perfect health) to one (worst possible health state) and LE was obtained from the actuarial life tables.

Statistical analysis

The overall survival (OS) was defined by the number of days from the date of initial diagnosis until the date of death or the last contact. The censored cases included patients without death at the time of the last follow up. The Kaplan-Meier method was used to estimate the OS rates and the univariate significance of differences among survival curves was calculated by the log-rank test. The co-variables associated with the OS were determined by the multivariate Cox regression model. Hazards ratio (HR) was used to estimate the time-to-event outcome with associated 95% confidence intervals (CIs) and p-values ≤ 0.05 were considered statistically significant. Average QALYs gained and average DALYs saved were calculated for all the variables stratified by the tumor histology. The SPSS 24.0 software (IBM Corp., Armonk, NY, USA) was used for data analyses.

## Results

Patient characteristics

In this 85 NPC cohort, there were 55 (64.7%) male patients and 30 (35.3%) female patients. The median age was 56 years (range, 19-86 years). Out of the 85 NPC patients, 35 (41.2%) had WHO Type I disease, 36 (42.4%) had WHO Type II disease, and 14 (16.5%) had WHO Type III disease. Fifty-seven (67.1%) were insured while 28 (32.9%) had no insurance. More than half of the patients 60 (70.6%) had a history of smoking and the use of alcohol was 50-50 in this group. The stage distribution in this cohort was Stage I (5.9%), Stage II (9.4%), Stage III (16.5%), Stage IV (52.9%), and Unknown Stage (15.3%). Fifty-one (60%) were treated with concurrent chemoRT, 20 (23.5%) with induction chemo followed by the chemoRT, and 14 (16.5%) NPC patients were hospice (Table 1).

**Table 1:** Demographic and clinical description of the nasopharyngeal study population by histology - WHO classification WHO = World Health Organization; induction chemo = induction chemotherapy; chemoRT = chemoradiation therapy

Variable	WHO Type I	WHO Type II	WHO Type III	All patients	p-value
	n= 35 (41.2%)	n= 36 (42.4%)	n= 14 (16.5%)	n = 85 (100%)	
GENDER/SEX					
Male	24 (68.6%)	20 (55.6%)	11 (78.6%)	55 (64.7%)	
Female	11 (31.4%)	16 (44.4%)	3 (21.4%)	30 (35.3%)	0.256
AGE					
< 35 years	1 (2.9%)	5 (13.9%)	3 (21.4%)	9 (10.6%)	
35 - 60 years	22 (62.9%)	16 (44.4%)	6 (42.9%)	44 (51.8%)	
> 60 years	12 (34.3%)	15 (41.7%)	5 (35.7%)	32 (37.6%)	0.232
ETHNICITY					
Black	16 (45.7%)	22 (61.1%)	9 (64.3%)	47 (55.3%)	
White	17 (48.6%)	12 (33.3%)	4 (28.6%	33 (38.8%)	
Others	2 (5.7%)	2 (5.6%)	1 (7.1%)	5 (5.9%)	0.633
INSURANCE					
Medicaid	9 (25.7%)	11 (30.6%)	3 (21.4%)	23 (27.1%)	
Medicare	8 (22.9%)	7 (19.4%)	4 (28.6%)	19 (22.4%)	
Private	5 (14.3%)	8 (22.2%)	2 (14.3%)	15 (17.6%)	
Self-pay	13 (37.1%)	10 (27.8%)	5 (35.7%)	28 (32.9%)	0.921
DISTANCE					
< 30 miles	12 (34.3%)	7 (19.4%)	5 (35.7%)	24 (28.2%)	
30 - 75 miles	6 (17.1%)	8 (22.2%)	1 (7.1%)	15 (17.6%)	
> 75 miles	17 (48.6%)	21 (58.3%)	8 (57.1%)	46 (54.1%)	0.499
SMOKING					
Smoker	29 (82.9%)	22 (61.1%)	9 (64.3%)	60 (70.6%)	
Non-Smoker	6 (17.1%)	14 (38.9%)	5 (35.7%)	25 (29.4%)	0.113
ALCOHOL					
Drinker	22 (62.9%)	14 (38.9%)	7 (50.0%)	43 (50.6%)	
Non-Drinker	13 (37.1%)	22 (61.1%)	7 (50.0%)	42 (49.4%)	0.130
TUMOR STAGE					
Stage I	2 (5.7%)	1 (2.8%)	2 (14.3%)	5 (5.9%)	
Stage II	3 (8.6%)	5 (13.9%)	0 (0.0%)	8 (9.4%)	
Stage III	6 (17.1%)	8 (22.2%)	0 (0.0%)	14 (16.5%)	
Stage IV	21 (60.0%)	19 (52.8%)	5 (35.7%)	45 (52.9%)	
Unknown Stage	3 (8.6%)	3 (8.3%)	7 (50.0%)	13 (15.3%)	0.005
TREATMENT					
Concurrent chemoRT	21 (60.0%)	24 (66.7%)	6 (42.9%)	51 (60.0%)	
Induction chemo + chemoRT	9 (25.7%)	8 (22.2%)	3 (21.4%)	20 (23.5%)	
Hospice	5 (14.3%)	4 (11.1%)	5 (35.7%)	14 (16.5%)	0.292

QoL data

The average QALYs gained and average DALYs saved for different variables was stratified by the histology of the NPC in the study population (Table 2). The average LE for this 85-patient NPC cohort was 34.51 years; average DALYs saved with treatment were 20.06 years and average QALYs gained with treatment were 11.77 years. The DALYs saved with concurrent chemoRT were 12.2 years *vs.* five years with induction chemo followed by concurrent chemoRT. The QALYs gained with concurrent chemoRT were 6.9 years *vs.* 3.1 years with induction chemo followed by concurrent chemoRT.

**Table 2:** QALYs and DALYs of the nasopharyngeal study population QALY = quality-adjusted life year; DALY = disability-adjusted life year; chemoRT = chemoradiation therapy; Induction chemo = induction chemotherapy

Variable	Avg. QALYs Gained	Avg. DALYs Saved
GENDER/SEX		
Male	11.63	20.19
Female	10.06	18.53
AGE		
< 35 years	17.01	26.01
35 - 60 years	12.35	21.37
> 60 years	7.67	15.38
ETHNICITY		
Black	11.53	20.09
White	11.51	19.62
Others	4.06	15.00
INSURANCE		
Medicaid	13.55	22.75
Medicare	8.67	15.77
Private	8.33	16.63
Self-pay	12.17	21.22
DISTANCE		
< 30 miles	11.49	20.73
30 - 75 miles	13.10	20.35
> 75 miles	10.21	18.78
SMOKING		
Smoker	10.86	19.43
Non-Smoker	11.62	20.04
ALCOHOL		
Drinker	11.03	19.84
Non-Drinker	11.13	19.36
TUMOR STAGE		
Stage I	7.71	15.67
Stage II	8.07	17.28
Stage III	11.20	18.52
Stage IV	12.40	21.27
Unknown Stage	9.49	17.95
TREATMENT		
Concurrent ChemoRT	10.84	19.90
Induction Chemo + ChemoRT	12.61	20.22
Hospice	9.75	17.66

Survival outcomes

**Figure 1:**
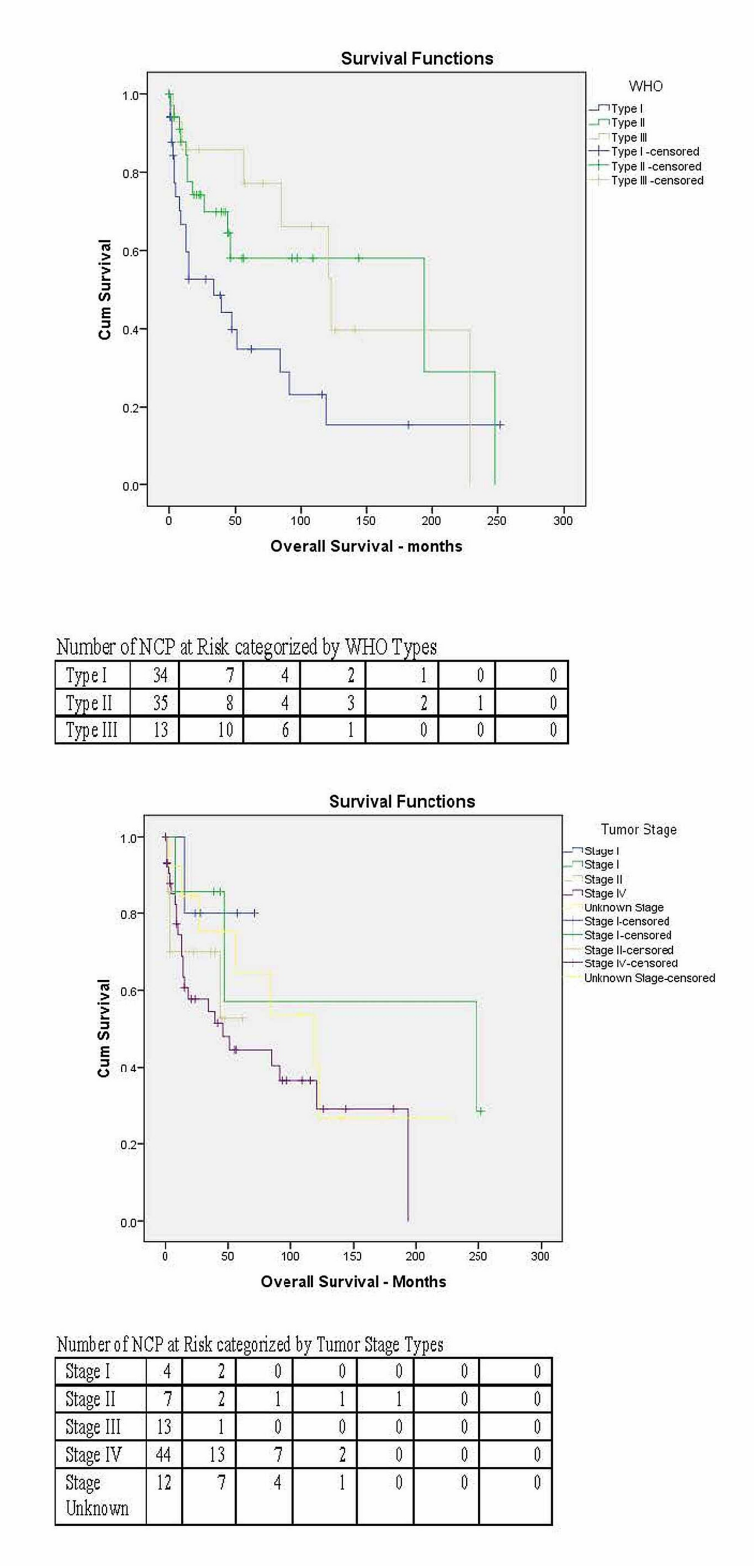
Kaplan-Meier overall survival curves for the study population by tumor histology (WHO types) and tumor stages WHO = World Health Organization; NCP = nasopharyngeal carcinoma patients

The median follow up for this study population was 24 months. The five-year OS of WHO Type I was 34.8%, Type II 58%, and Type III 77.1% (p=0.042). The median survival of WHO Type I was 34 months, Type II 194 months, and Type III 123 months (Figure 1). The OS curve for different tumor stages are represented in Figure 1. For all histologies, the five-year OS for Stage I was 80.0%, Stage II 57.1%, Stage III 52.6%, Stage IV 44.5%, and Stage Unknown 64.5% (p=0.321) .

Univariate analyses

**Figure 2:**
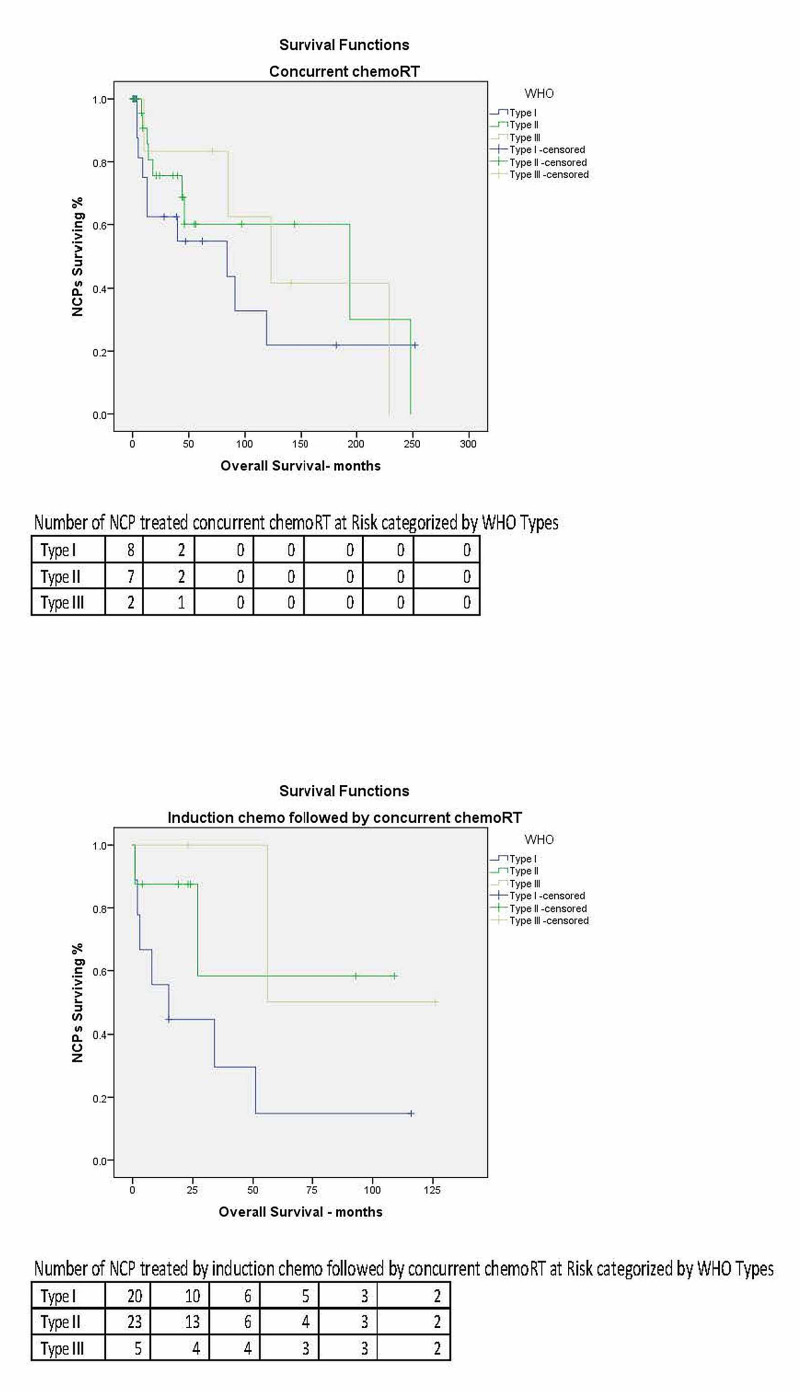
The Kaplan-Meier overall survival curves for NCPs treated with concurrent chemoRT vs. NCPs treated with induction chemo followed by concurrent chemoRT WHO = World Health Organization; NCP = nasopharyngeal carcinoma patients; chemoRT= chemoradiation therapy; induction chemo = induction chemotherapy

In the univariate analyses, the variables of gender, age, ethnicity, insurance, distance traveled to the treatment facility, alcohol history, and the treatment modality were significantly associated with the OS (Table 3). The OS curves for the treatment modalities used are represented in Figure 2. Five-year OS of NCPs treated by concurrent chemoRT for WHO Type I was 54.7%, WHO Type II 60.1%, and WHO Type III 83.3 % (p= 0.029). Five-year OS of NCPs treated by induction chemo followed by concurrent chemoRT for WHO Type I was 14.8 %, WHO Type II 58.3%, and WHO Type III 50.0% (p=0.029).

**Table 3:** Univariate analysis by tumor histology

Variable	log rank p-value
Gender/sex	0.013
Age	0.013
Ethnicity	0.042
Insurance	0.026
Distance	0.041
Smoking	0.960
Alcohol	0.010
Treatment	0.029

Multivariate analyses

The variables which were significantly associated with survival outcomes were included in the Cox proportional hazards regression model (Table 4). In the multivariate analyses, only tumor histology was significantly associated with OS, with HR of 0.61 (95% CI 0.39-0.94), p= 0.027.

**Table 4:** Multivariate Cox regression analysis HR = hazard ratio; CI = confidence interval

Variable	HR (95% CI)	p-value
Gender/sex	0.52 (0.24 - 1.15)	0.111
Age	0.84 (0.48 - 1.47)	0.557
Ethnicity	0.87 (0.50 - 1.53)	0.879
Insurance	1.13 (0.85 - 1.49)	0.389
Distance	0.87 (0.58 – 1.29)	0.496
Alcohol	1.40 (0.71 – 2.78)	0.324
Treatment	0.88 (0.48 – 1.59)	0.682
Tumor histology	0.61 (0.39 – 0.94)	0.027

## Discussion

Nasopharyngeal carcinoma is a rare neoplasm of the HN in the United States and accounts for only 2% of all the HN cancers [1,2]. Our data for this cohort of patients, accounting for 2.4% of all the patients in our HN cancer database, is consistent with the literature. Patients with NPC are mostly males with a known history of tobacco and heavy alcohol use [19], and our data reflects what has been previously reported as 65% were males with 71% had a known history of tobacco and 51% had a history of heavy alcohol use. Nasopharyngeal carcinoma is more common in Asians/Pacific Islanders, who are diagnosed six times more frequently than Caucasian and Hispanic people [20], and in whom the disease can occur at any age, including children, while about 50% of people with the disease are 55 years or younger. Our patient cohort consisted of 39% Caucasian and 55 % African Americans, which is most likely due to our regional demographics, while 53% of the cohort were younger than 60 years.

Generally, the NPC histology distribution in the United States is keratinizing carcinoma (Type I) 25%, differentiated non-keratinizing (Type II) 12%, and undifferentiated non-keratinizing (Type III) 63%. In our cohort, these histologies, however, were represented by 41%, 42%, and 17%, respectively. It is possible that these differences are due to the ethnic demographics of our patient population, with very few Asian/Pacific Islanders represented. In the literature, the reported five-year OS for non-keratinizing NPC is 51% *vs.* 6% for keratinizing NPC [10]. In our cohort though, the five-year OS was 66% *vs.* 38%, with a median survival of 24 months.

It is established that NPC patients have a higher likelihood of living cancer-free for an extended period of time with treatment methods that utilize radiation therapy (RT) alone or in combination with chemotherapy [21]. Many reports have suggested that treatments delivered with more advanced RT techniques show a significant trend toward improving QoL outcomes [22, 23]. In a prospective randomized trial comparing the QoL of NPC patients treated with two-dimensional radiation therapy (2DRT) *vs.* intensity-modulated radiotherapy (IMRT), it was shown that IMRT significantly improved salivary flow after RT, although no other scale being measured significantly improved, except physical and role functioning, as assessed by the EORTC QLQ-C30 [24]. Some studies have noted that the use of three-dimensional chemoRT/IMRT reduced the HN-related symptom scales to some extent and thereby improved QoL [25]. Besides the RT technique used, socioeconomic status, comorbidity, and tumor site were also found to be significant prognostic predictors of a patient’s HR-QoL outcome. Patients who had higher economic status, higher education levels, employed status, and fewer comorbidities tended to have better HR-QoL [26]. Some studies have reported that female gender, higher cancer stage, and combination treatment were associated with more symptoms and worse HR-QoL [27, 28]. Few studies had explored the prognostic significance of QoL in QLQ-C30 questionnaires for NPC patients. Studies conducted to evaluate the impact of replanning on the QoL during IMRT showed that replanning had both statistically and clinically significant improvements in QoL scales [29]. A study by Tan et.al. [30] indicates that induction chemo followed by chemoRT was not shown to improve OS compared to definitive chemoRT. In our study, we found that the OS of concurrent chemoRT *vs.* induction chemo followed by concurrent chemoRT was 32.8% *vs.* 14.8% (p=0.029).

Many studies have previously used the EORTC health survey questionnaires to investigate the QoL of NPC patients. For our analysis, however, we were unable to estimate patient QoL using the questionnaire method as our institution started using health survey questionnaires only recently. Thus, in order to calculate QoL without available survey data, we used QALYs and DALYs as health outcome measures that account for both longevity and QoL [16]. In our investigation, we found that the average LE for our 85 NPC cohort was 34.56 years, the average DALYs saved with treatment were 20.06 years, and the average QALYs gained with treatment were 11.77 years. The DALYs saved with concurrent chemoRT were 12.2 years *vs.* five years with induction chemo followed by concurrent chemoRT. The QALYs gained using concurrent chemoRT were 6.9 years *vs.* 3.1 years with induction chemo followed by concurrent chemoRT.

As with all retrospective analyses, our study is not without its limitations. As we had no HR-QoL data available, potential selection bias is one of the limitations. The QALY approach used in this study does not explicitly incorporate equity weights, which can be a challenge when comparing treatment interventions. In order to overcome the challenges posed by using the QALY approach, we utilized an alternative method, DALY, to compare the impact of disease burden among treatment modalities.

## Conclusions

Our retrospective analysis reports OS findings in NPC similar to that in previous studies, indicating that induction chemo followed by chemoRT was not shown to improve OS compared to definitive chemoRT. In addition, our data suggest that patients treated with concurrent chemoRT had an improved QoL compared to that of patients treated with induction chemo followed by concurrent chemoRT. The average DALYs saved and average QALYs gained were higher in the patients treated with concurrent chemoRT. However, there may be some yet unidentified factors or some specific functional domains related to the QoL which were unappreciated in this analysis.
